# Thickness-dependent photoelectric properties of MoS_2_/Si heterostructure solar cells

**DOI:** 10.1038/s41598-019-53936-2

**Published:** 2019-11-22

**Authors:** Yipeng Zhao, Gang Ouyang

**Affiliations:** Key Laboratory of Low-Dimensional Quantum Structures and Quantum Control of Ministry of Education, Synergetic Innovation Center for Quantum Effects and Applications (SICQEA), Hunan Normal University, Changsha, 410081 China

**Keywords:** Materials science, Materials for energy and catalysis

## Abstract

In order to obtain the optimal photoelectric properties of vertical stacked MoS_2_/Si heterostructure solar cells, we propose a theoretical model to address the relationship among film thickness, atomic bond identities and related physical quantities in terms of bond relaxation mechanism and detailed balance principle. We find that the vertical stacked MoS_2_/Si can form type II band alignment, and its photoelectric conversion efficiency (PCE) enhances with increasing MoS_2_ thickness. Moreover, the optimal PCE in MoS_2_/Si can reach 24.76%, inferring that a possible design way can be achieved based on the layered transition metal dichalcogenides and silicon.

## Introduction

Two-dimensional transition metal dichalcogenides (2D-TMDs) have emerging as promising candidates in optoelectronic devices owing to their intriguing properties, such as strong electron-hole confinement, as well as excellent mechanical and thermal stability^1–3^. Typically, molybdenum disulfide (MoS_2_), a member of TMD, possesses strong light-matter interactions and outstanding absorption ability in the range of visible light region, generating impressive applications in photovoltaics^4–6^. Besides, MoS_2_ shows considerable carrier mobility of ~200 cm^2^ V^−1^ s^−1^ for monolayer and ~500 cm^2^ V^−1^ s^−1^ for multi-layers^7,8^. Meanwhile, the weak interlayer van der Waals (vdW) interactions enable large area and uniform atomic layers of MoS_2_ to be isolated, and the elimination of dangling bonds is beneficial to form heterostructures^9,10^.

In general, the successful growths of monolayer and few-layer MoS_2_ have provoked the fabrication of MoS_2_-based electronic nanodevices^3,7,9,11^. Moreover, Si is the dominating electronic material due to its high abundance and mature processing technology. Thus, it is meaningful to realize the integration of MoS_2_ on Si to develop practically applicable solar cells. Currently, some observations have shown that the novel photoelectric properties of MoS_2_/Si^11–14^. For example, Tsai *et al*.^11^ reported a photoelectric conversion efficiency (PCE) of high-quality monolayer MoS_2_ on Si substrate is 5.23%. Lopez-Sanchez *et al*.^12^ found that the vertical MoS_2_/Si heterostructure has an external quantum efficiency of 4.4% and expresses a broad spectral response. In nature, layered MoS_2_/Si heterostructure establishes a built-in electronic field at the interface that helps in carrier separation for photovoltaic operation^15–17^. Moreover, the passivation of surface and interface in solar cells will enhance the photovoltaic behavior due to the integration of efficient charge carrier separation/isolation mechanism^18,19^. Therefore, the heterostructure composed of Si and layered materials preserve the complementary advantages of both components, providing an innovative approach to construct high-performance optoelectronic devices.

In spite of several achievements with the photoelectric properties of MoS_2_/Si heterostructure, a systematic study to illustrate the thickness dependence of PCE is still lacking. Fundamentally, some problems should be clarified urgently at the atomic-level, including how to quantify the carrier diffusion and collection, and how to realize the optimized configurations. Therefore, in this contribution, we establish an analytical method to investigate the influence of bonding parameters on the band alignment and PCE of MoS_2_/Si heterostructure in terms of atomic-bond-relaxation (ABR) consideration^20–23^ and detailed balance principle (DBP)^24,25^. Our method provides a reliable and useful way for gaining insight into the transport mechanism and photoelectric properties of two-dimensional (2D)/three-dimensional (3D) heterostructures, suggesting a helpful guidance for both fundamental investigation and device design.

In general, with the shrinking of thickness, the role of surface and interface becomes more and more important. According to ABR mechanism, the abrupt termination of bonding network can leave a high dangling bond and coordination deficiency in the end parts^26,27^. Thus, the system will be in a self-equilibrium state and the strain will be occurrence, which makes some relevant quantities such as electronic density and binding energy distinctive from their corresponding bulk^20,28^. Notably, the bond strain can be expressed as: $\varepsilon ={d}^{\ast }/{d}_{0}-1$, where d^*^ and *d*_0_, respectively, denote the average bond length and that of the bulk. Considering the discrepancy between the surface and core interior, the average strain can be deduced as: $\langle \varepsilon \rangle =\sum _{i < {n}_{s}}{\gamma }_{ic}({z}_{i}{c}_{i}/{z}_{b}-1)$, where *n*_*s*_ is the number of surface layers, *z*_*i*_ and *z*_*b*_ are the effective coordination numbers (CNs) of specific *i*th atomic layer and that of the bulk, ${\gamma }_{ic}=\sum _{i\le {n}_{s}}4{c}_{i}{d}_{0}/D$ is surface-to-volume ratio (SVR), where ${c}_{i}=2/\{1+\exp [(12-{z}_{i})/8{z}_{i}]\}$ is the bond contraction coefficient, *D* is the film thickness^20,21,29^.

It should be noted that there are some types of procedures for fabrication of MoS_2_/Si heterostructure, such as the synthesis of MoS_2_ and subsequent transfer to Si substrate or direct growth of MoS_2_ on Si, etc^11–14^. The large difference of lattice structure would lead to alternating compressive and tensile strains at the interface, resulting different electronic structure and physical properties^30–33^. For instance, Scheuschner *et al*.^31^ prepared MoS_2_ layers via mechanical exfoliation of natural MoS_2_ on Si/SiO_2_ substrates, and observed the photoluminescence peak (PL) of MoS_2_ shows a red-shift of ~65 meV. Liu *et al*.^32^ found that the tensile strain in MoS_2_ is released after transfer MoS_2_ to Si/SiO_2_ substrate, and the global tensile strain is estimated to be 1%. Besides, the interface strain can be induced by the type of substrate and post heating/cooling of the 2D material-substrate system, etc.

**Figure 1:**
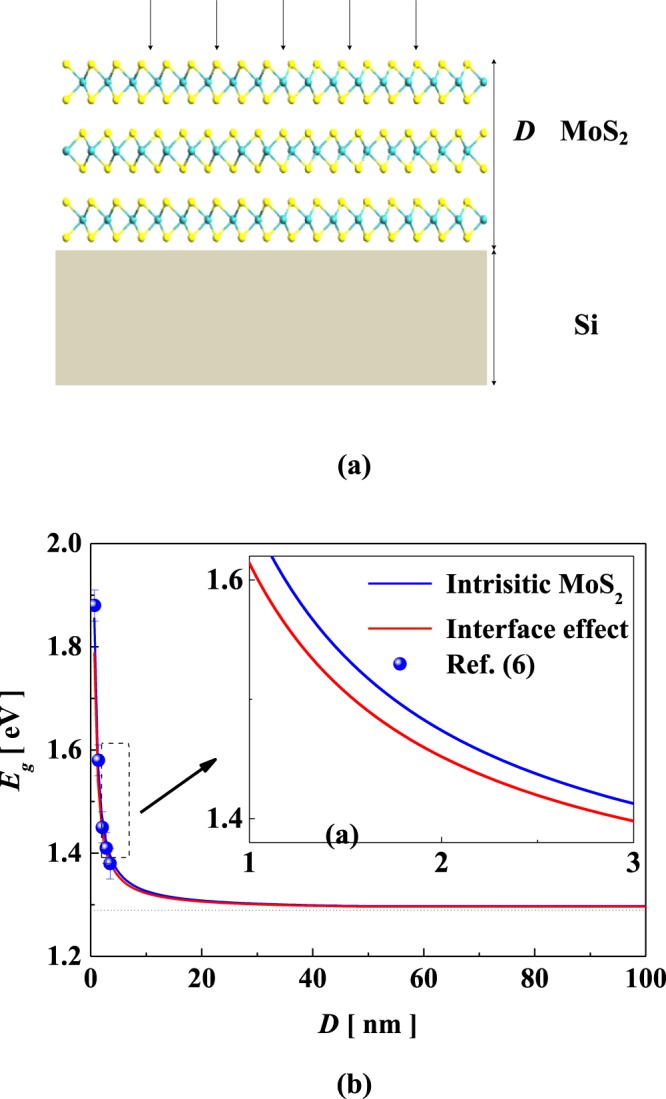
(**a**) The schematic diagram of a vertical stacked MoS_2_/Si. (**b**) Thickness-dependent bandgap of MoS_2_. The thickness of monolayer MoS_2_ is 0.65 nm, and the Mo-S bond length is 2.41 Å.

In our case, we construct a prototype of vertical stacked MoS_2_/Si shown in Fig. 1a. Noticeably, the interaction energy at the interface composed of vdW interaction energy and interface strain energy. In general, the vdW interaction can be characterized as: $U=\frac{1}{2}{\sum }_{i=1}^{n}{\sum }_{j=1}^{n}u(r)$, with $u(r)=-\,\Gamma [{(\sigma /r)}^{12}-{(\sigma /r)}^{6}]$, where *i* and *j* represent the atom *i* and *j*_,_ Γ and σ are the constants for the attractive and repulsive interaction^34^. Ignore the influence of dislocation formation, the mismatched strain is: ${\varepsilon }_{m}=({a}_{S{\rm{i}}}-{a}_{M{\rm{o}}})/{a}_{Mo}$, where *a*_*Si*_ and *a*_*Mo*_ are the lattice constants of Si and MoS_2_, respectively. Thus, the compatibility of the deformation can be written as: *ε*_*Mo*_ − *ε*_*M*_ = *ε*_*Si*_, where *ε*_*Mo*_ and *ε*_*Si*_ are the mean elastic extensional strain in the MoS_2_ and Si, respectively. Notably, the net force on any internal plane perpendicular to the interface must be zero under the condition of self-equilibrium state, obeying

1.1 $${E}_{Mo}{\varepsilon }_{Mo}D+{E}_{Si}{\varepsilon }_{Si}{D}_{Si}=0$$

where *E*_*Mo*_ and *E*_*Si*_ are the Young’s moduli of MoS_2_ and Si, and *D*_*Si*_ is the thickness of Si, respectively^35^.

Naturally, the interaction potential of MoS_2_ can be expressed as the summation of bond-stretching energy *E*_*bond*_, the bond angle variation energy *E*_*angle*_ and the Coulomb electrostatic energy *E*_*c*_^36,37^, i.e.,

1.2 $${E}_{M}=\sum {E}_{{bond}}+\sum {E}_{{angle}}+\sum {E}_{{c}}$$

where ${E}_{{bond}}={D}_{b}\times {[1-{e}^{-\alpha {({h}_{ij}-h)}^{2}}]}^{2}$, ${E}_{{angle}}=\frac{1}{2}{k}_{\theta }{(h\Delta \theta )}^{2}+\frac{1}{2}{k}_{\psi }{(h\Delta \psi )}^{2}$ and *E*_*c*_ = *C*·*q*_*i*_*q*_*j*_/*h*_*ij*_, where *D*_*b*_, *α*, *k*_*θ*_, *k*_*ψ*_ are the bond potential parameters and *C* is the Coulomb electrostatic potential parameter, *h*_*ij*_ is the distance between atoms *i* and *j*, Δ*θ* and Δ*ψ* are the changes of in-plane and out-of-plane bond angles, *q*_*i*_ and *q*_*j*_ are the partial electrostatic charges for atoms *i* and *j*^37^.

Considering the joint effect from the surface and interface, the cohesive energy is

1.3 $${E}_{C}=\sum _{i\le {n}_{s}}[{N}_{i}{z}_{i}{E}_{i}+{N}_{{int}}{z}_{{int}}{E}_{{int}}+(N-{N}_{i}-{N}_{{int}}){z}_{b}{E}_{b}]$$

where *N*_*i*_ and *N*_*int*_ denote the atomic numbers of the specific *i*th atomic layer and interface layer, *N* is the total numbers, *z*_*i*_(*E*_*i*_), *z*_*int*_(*E*_*int*_) and *z*_*b*_(*E*_*b*_) are the effective CNs (single bond energy) of the spec*i*fic *i*th atomic layer, interface layer and that of the bulk. Remarkably, the bond order loss of an atom in the surface and interface causes the remaining bonds of the under-coordinated atom to contract spontaneously, leading to the intra-atomic potential well depression from *E*_*b*_ to ${E}_{i}={c}_{i}^{-m}{E}_{b}$, where *m* is the bond nature factor^20,21^.

Furthermore, the bandgap is from the crystal potential over the entire solid, and the bandgap is proportional to the first Fourier coefficient of the crystal potential, which is in proportional to the single bond energy, i.e., ${E}_{g}\propto \langle {E}_{0}\rangle ={E}_{C}/\langle z\rangle N$, where $\langle z\rangle =\sum _{i\le {n}_{s}}{\gamma }_{i}({z}_{i}-{z}_{b})+{\gamma }_{{int}}({z}_{i}-{z}_{b})+{z}_{b}$ is the average CNs^29^. Consequently, the thickness-dependent bandgap of MoS_2_ can be obtained

1.4 $$\frac{{E}_{g}}{{E}_{g}^{b}}=\frac{\sum _{i\le {n}_{s}}{\gamma }_{i}({z}_{i}{E}_{i}-{z}_{b}{E}_{b})+\sum {\gamma }_{{int}}({z}_{{int}}{E}_{{int}}-{z}_{b}{E}_{b})+{z}_{b}{E}_{b}}{\langle z\rangle {E}_{b}}$$

where ${E}_{g}^{b}$ is the bandgap of the bulk counterpart.

Moreover, the shifts of conduction band minimum (CBM) and valence band maximum (VBM) is tightly related to the effective mass of electron and hole^38,39^. Therefore, the shifts of CBM and VBM are

15 $$\{\begin{array}{rcl}\Delta {E}_{{\rm{CBM}}} & = & \Delta {E}_{g}\frac{{m}_{h}^{\ast }}{{m}_{e}^{\ast }+{m}_{h}^{\ast }}\\ \Delta {E}_{{\rm{VBM}}} & = & \Delta {E}_{g}\frac{{m}_{e}^{\ast }}{{m}_{e}^{\ast }+{m}_{h}^{\ast }}\end{array}$$

where ${m}_{e}^{\ast }$ and ${m}_{h}^{\ast }$ are the effective masses of electron and hole, respectively.

Also, the energy band alignment of heterostructure plays a critical role for determining the electronic properties. In the case of semiconductor-semiconductor interface, the conduction band offset (CBO) Δ*E*_*c*_ and valance band offset (VBO)Δ*E*_*v*_ at the interface are shown as

1.6 $$\{\begin{array}{rcl}\Delta {E}_{{c}} & = & {\chi }_{1}-{\chi }_{2}\\ \Delta {E}_{{v}} & = & \Delta {E}_{g}-\Delta {E}_{{c}}\end{array}$$

where *χ*_1_ and *χ*_2_ are the electron affinity of MoS_2_ and Si, respectively. In the case of MoS_2_/Si heterostructure, the width of space charge region is mainly determined by the concentration of carriers, i.e.,

1.7 $${X}_{{scr}}=\sqrt{\frac{2{\varepsilon }_{1}{\varepsilon }_{2}({({N}_{d}+{N}_{a})}^{2}{V}_{bi})}{q{N}_{a}{N}_{d}({\varepsilon }_{1}{N}_{d}+{\varepsilon }_{2}{N}_{a})}}$$

where *ε*_1_ and *ε*_2_ denote the relative permittivity of MoS_2_ and Si, *N*_*d*_ and *N*_*a*_ are the ion doping concentration of MoS_2_ and Si. *V*_*bi*_ is the built-in potential that can be deduced as: ${V}_{bi}=\Delta {E}_{{\rm{c}}}+kT(\frac{{N}_{c}{N}_{v}}{{N}_{d}{N}_{a}})$, where *N*_*c*_ and *N*_*v*_ represent the effective conduction band density of MoS_2_ and effective valence band density of Si, respectively^40^. Thus, the widths of space charge region in MoS_2_ and Si are: ${X}_{n}=\frac{{N}_{a}}{{N}_{a}+{N}_{d}}{X}_{{scr}}$ and ${X}_{p}=\frac{{N}_{d}}{{N}_{a}+{N}_{d}}{X}_{{scr}}$.

On the other hand, the short current density is determined by the absorptance that can be obtained through the absorption coefficient and thickness. Generally, the absorptivity of solar radiation in the heterostructure is

1.8 $$\{\begin{array}{rcl}{A}_{1}(v) & = & (1-R)\cdot 1-{e}^{(-{\alpha }_{1}(v)D)}\\ {A}_{2}(v) & = & (1-R)\cdot {e}^{(-{\alpha }_{1}(v)D)}\cdot (1-{\alpha }_{2}(v){D}_{Si})\end{array}$$

where *v* is the photon frequency, *R* is the reflectance of incident surface, and ${\alpha }_{{m}^{\#}}(v)$ $({m}^{\#}=1,\,2)$ is the absorption coefficient of MoS_2_ and Si, respectively. The reflectivity of interface for normal incidence is $R={(1-\langle n\rangle )}^{2}/{(1+\langle n\rangle )}^{2}$, where $\langle n\rangle ={n}_{2}/{n}_{1}$ denotes the relative refractive index of interface^41^.

Noticeably, the absorption coefficient for a given photon energy is proportional to the probability for the transition from the initial state *i* to the final state *f* and to the occupied state density of electrons in the initial state, ${n}_{i}({E}_{i})$, and also to the unoccupied state density of final states, ${n}_{f}({E}_{f})$, i.e., $\alpha (\nu )\propto \sum _{i,f}{W}_{i,f}{n}_{i}({E}_{i}){n}_{f}({E}_{f})$, where *W*_*i,f*_ is the transition probability. In the case of indirect interband transition, a two-step process is indispensable because the photon cannot provide a change in momentum. Hence to complete the transition, a phonon can either be absorbed or emitted to conserve the momentum of the electrons. The phonon and photon energy satisfies: *hv*_*e*_ = *E*_*f*_ − *E*_*i*_ + *E*_*p*_ for the phonon emission and *hv*_*e*_ = *E*_*f*_ − *E*_*i*_ − *E*_*p*_ for phonon absorption, where *E*_*p*_ is the phonon energy. The number of phonons is given by Bose-Einstein statistics: ${n}_{p}=\frac{1}{\exp ({E}_{p}/{k}_{0}T)-1}$^42^. Additionally, in the case of indirect transitions, all the occupied states of the valence band can connect to all the empty states of the conduction band. Thus, the density of initial and final states is

1.9 $$\{\begin{array}{rcl}{n}_{i}({E}_{i}) & = & \frac{1}{2{\pi }^{2}{\hslash }^{3}}{(2{m}_{h}^{\ast })}^{3/2}{|{E}_{i}|}^{1/2}\\ {n}_{f}({E}_{f}) & = & \frac{1}{2{\pi }^{2}{\hslash }^{3}}{(2{m}_{e}^{\ast })}^{3/2}{({E}_{f}-{E}_{g})}^{1/2}=\frac{1}{2{\pi }^{2}{\hslash }^{3}}{(2{m}_{e}^{\ast })}^{3/2}{(h\nu -{E}_{g}\mp {E}_{p}-{E}_{i})}^{1/2}\end{array}$$

Accordingly, the absorption coefficient for a transition with phonon absorption can be shown as

1.10 $$\{\begin{array}{rcl}{\alpha }_{{m}^{\#}}(v) & = & {A}_{m}[\frac{{(h\nu -{E}_{g}+{E}_{p})}^{2}}{\exp ({E}_{p}/{k}_{0}T)-1}+\frac{{(h\nu -{E}_{g}-{E}_{p})}^{2}}{1-\exp (-{E}_{p}/{k}_{0}T)}]\,\,\,hv > {E}_{g}+{E}_{p}\\ {\alpha }_{{m}^{\#}}(v) & = & {A}_{m}\frac{{(h\nu -{E}_{g}+{E}_{p})}^{2}}{\exp ({E}_{p}/{k}_{0}T)-1}\,\,\,{E}_{g}-{E}_{p} < hv\le {E}_{g}+{E}_{p}\end{array}$$

where *A*_*m*_ is the material constant.

Moreover, the differential equation of excess minority carrier density is given by

1.11 $$\{\begin{array}{l}\frac{{d}^{2}{n}_{p}(x)}{d{x}^{2}}-\frac{{n}_{p}(x)-{n}_{p0}}{{L}_{n}^{2}}+\frac{1}{{D}_{n}}\int G(x,\nu )d\nu =0\\ \frac{{d}^{2}{p}_{n}(x)}{d{x}^{2}}-\frac{{p}_{n}(x)-{p}_{n0}}{{L}_{p}^{2}}+\frac{1}{{D}_{p}}\int G(x,\nu )d\nu =0\end{array}$$

where *L*_*n*_ and *L*_*p*_ denote the diffusion lengths of electron and hole, respectively, *G* is the concentration of photon generated carriers. Generally, the diffusion length of carriers is determined by the diffusion coefficient *D*_*n,p*_ and life time *τ*_*n,p*_ of minority carrier, i.e., ${L}_{n}=\sqrt{{D}_{n}{\tau }_{n}}$, and ${L}_{p}=\sqrt{{D}_{p}{\tau }_{p}}$. In terms of Einstein equation, the diffusion coefficients are ${D}_{n}=\mu {}_{n}k_{B}T/q$ and ${D}_{p}=\mu {}_{p}k_{0}T/q$, where *k*_*B*_ denotes the Boltzmann’s constant, *T* is the absolute temperature, *μ*_*n*_ and *μ*_*p*_ are the carrier mobility of electron and hole, respectively^43^.

Actually, the carrier mobility can be separated into several parts: $1/\mu =1/{\mu }_{0}+1/{\mu }_{1}+\ldots +1/{\mu }_{k}$, where *μ*_0_ is the intrinsic carrier mobility, *μ*_*i*_ (*i* = 1 … *k*) is the contributions of phonon scattering, surface roughness scattering, interface effects and so on. Generally, the phonon scattering is *μ*_*ph*_∝*D*^2^m^*−1.5^, and surface roughness scattering is *μ*_*sr*_∝*μ*_*ph*_/Δ^2^∝*D*^2^m^*−1.5^/Δ^2^, where Δ is the root mean square roughness^44–46^. Consequently, the carrier mobility can be expressed as:

1.12 $${\mu }_{n,p}=\frac{{\mu }_{n,p}^{0}}{1+(A+B{\Delta }^{2}){\mu }_{n,p}^{0}{E}_{g}^{1.5}/{D}^{2}+{C}_{0}{E}_{g}^{0.5}D}$$

where *A*, *B*, *C*_0_ is the constant^47^.

Furthermore, the surface and interface passivation can enhance the photovoltaic behavior due to the integration of efficient charge carrier separation/isolation mechanism^18,19^. For instance, the passivation of MoS_2_ surface with Al_2_O_3_ dielectric layer has been demonstrated to enhance the PCE from 2.21% to 5.6% in multilayer MoS_2_/Si solar cells^19^. Physically, the surface passivation can effectively suppress the surface recombination. Consider the recombination contributions from the bulk and surface, the effective carrier lifetime is^48^

1.13 $$\frac{1}{{\tau }_{eff}}=\frac{1}{{\tau }_{b}}+\frac{4S}{D}$$

where *τ*_*b*_ is the carrier lifetime in the bulk case, *S* is the recombination velocity.

## Results

### Band shift and band alignment

Figure 1a depicts the schematic diagram of a vertical stacked MoS_2_/Si, and *D*(*D*_*Si*_) denotes the thickness of MoS_2_ (Si). Generally, the thickness of Si is micron scale and possesses bulk like properties. Figure 1b shows the evolution of bandgap with thickness of MoS_2_. Clearly, the bandgap increases monotonically with reducing thickness, and shows an obviously leap when the thickness shrinks down to a few nanometers. Taking into account the interface effect, the bandgap of MoS_2_ slightly decrease compared to that of the intrinsic case. Evidently, the ratio of interface and surface atoms increases with decreasing thickness, and the bond-order loss and CNs imperfection at surface and interfaces will lead the system relax to new self-equilibrium state, resulting in the change of Hamiltonian and related physical properties^20,21^. Similarly, Mak *et al*.^6^ found that the bandgap of layered MoS_2_ possesses obvious blue-shift from 1.2 eV to 1.9 eV with thickness reducing to monolayer. In addition, the lateral size, temperature and substrate can effective modulate the optical and electronic properties of MoS_2_. Mukherjee *et al*.^49,50^ has firstly investigated the evolution of optical properties of MoS_2_ nanocrystals with lateral size, and observed the direct bandgap transition in monolayer and few-layers of MoS_2_. The PL peaks are gradually blue-shifted with decreasing lateral size due to quantum confinement effect, demonstrating the potential of MoS_2_-based heterostructure for photoelectric devices.

**Figure 2:**
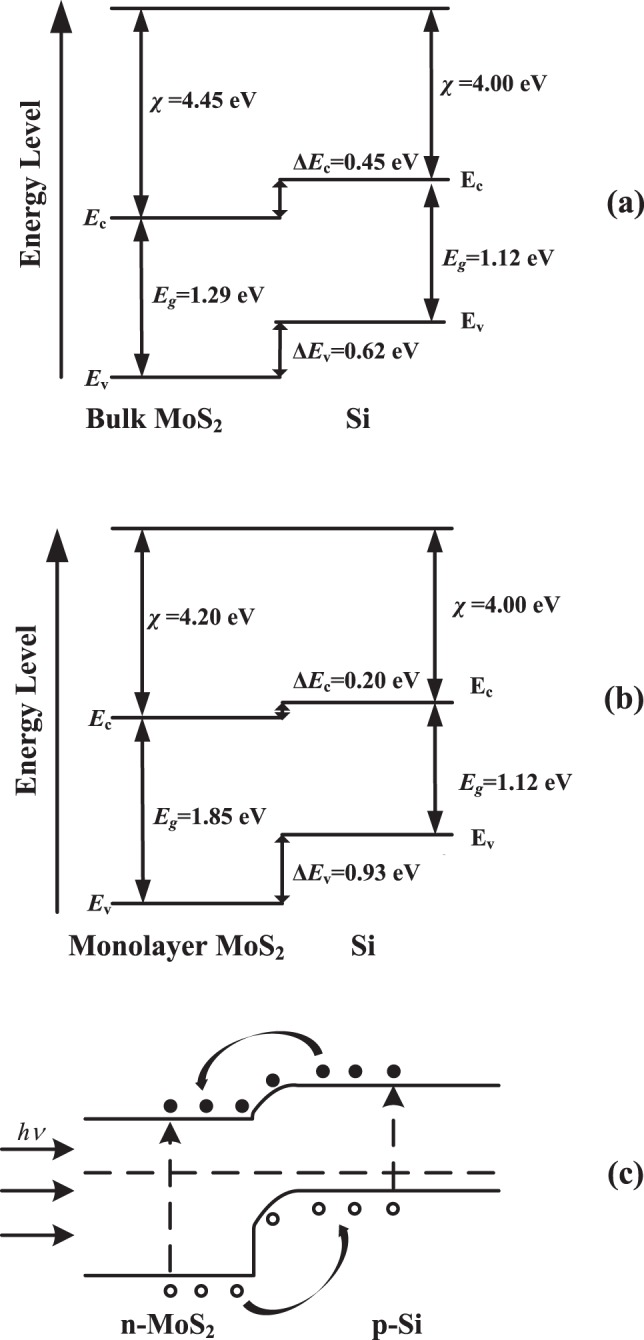
The band alignment of MoS_2_/Si heterostructure. (**a**) Bulk MoS_2_/Si, (**b**) Monolayer MoS_2_/Si, (**c**) Schematic of band diagram.

The band alignment of MoS_2_/Si is shown in Fig. 2. Note that *χ*_MoS2_ ~ 4.2 eV, and ~4.0 eV for Si in our calculation^51,52^. In the Fig. 2, we can see that the vertical stacked MoS_2_/Si heterostructure possesses type II band alignment with CBM located at the MoS_2_ layer and VBM at the Si part. In detail, the CBO is 0.45 eV for bulk like MoS_2_/Si and 0.20 eV for monolayer MoS_2_/Si. In nature, the built-in field at interface facilitates the separation of photo-generated electron-hole pairs, depressing the interlayer recombination and benefit the collection of free carriers. The photo-induced electrons are preferred to stay at MoS_2_ layer while holes prefer stay at Si layer (Fig. 2c). It is worth noting that interlayer recombination is the dominant recombination mechanism for ultrathin films, thus the rapid separation of carriers can drastically reduce the interface recombination. Interestingly, it can be inferred that the excellent light absorption and type II band alignment make the MoS_2_/Si possess fascinating application in solar cells.

### Thickness-dependent carrier mobility and diffusion length

**Figure 3:**
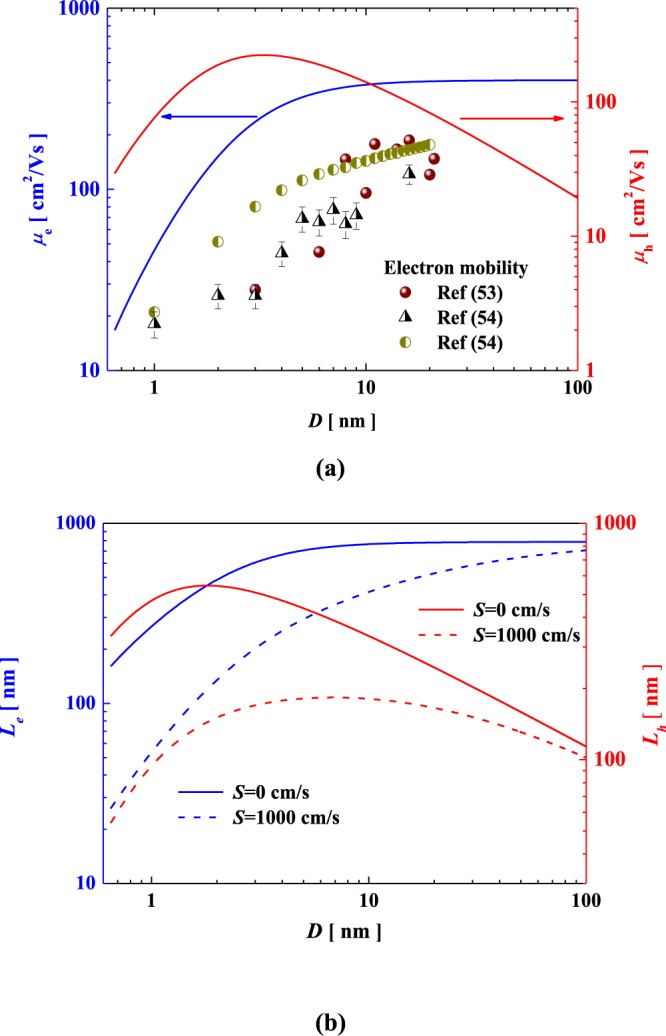
Thickness-dependent mobility (**a**) and diffusion length (**b**) of electron and hole in MoS_2_ films.

In Fig. 3a, we can see that the electron mobility increases monotonically with increasing thickness. Similarly, several experiments and calculations indicated that the mobility increases from 20 to 110 cm^2^·V^−1^·s^−1^ rapidly as the MoS_2_ layers enhances, and can be up to the bulk value beyond ~10 nm^53,54^. However, the hole mobility exhibits a first-rapid increase and then reduces with enhancing thickness, and reaches the maximum beyond ~3 nm. In fact, the phonon and surface roughness scattering determine the mobility for the few-layer MoS_2_, while the effect of subbands plays the vital role for the thick films^45,47^.

Figure 3b shows the thickness-dependent electron and hole diffusion lengths of MoS_2_. Clearly, as the thickness increases, the electron diffusion length increases monotonically and sharply, while that of hole increases initially and then decreases. In addition, it is clearly that the surface recombination reduces the diffusion length significantly, and the reduction becomes more obvious as the thickness decreases. Actually, a lot of factors such as impurity density, doping density and dielectric environment will influence the minority carrier mobility and diffusion length. Currently, the carrier motilities are limited by the impurity scattering, leading to the lower collection efficiency. Thus, it is important to explore suitable method to improve the carrier mobility since it is the dominant factor in the effective collection of free carriers and short current.

## Discussion

**Figure 4:**
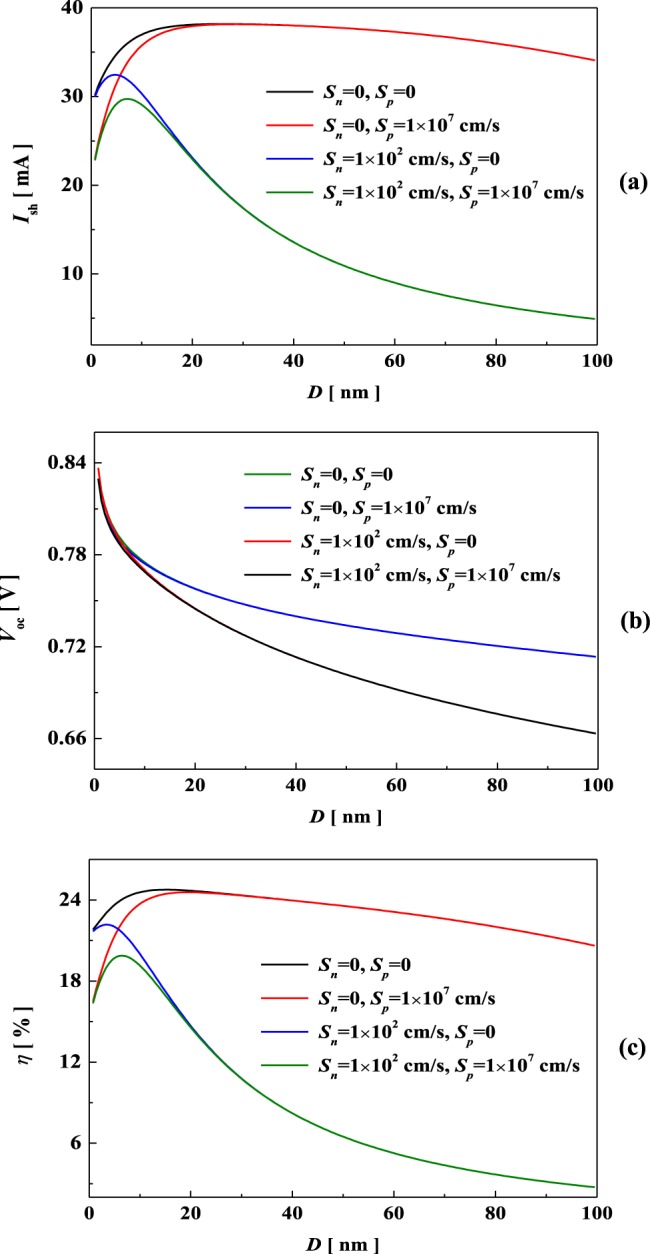
Thickness-dependent *I*_*sc*_(**a**), *V*_*oc*_(**b**), and PCE (**c**) in MoS_2_/Si solar cell.

Here we consider the photoelectric properties of MoS_2_/Si with varying thickness under the illumination condition of AM 1.5 solar irradiation. In our case, four different surface recombination (*S*_*n*_) and back surface recombination (*S*_*p*_) have been taken into account. As shown in Fig. 4a, the short current has evident thickness dependence. For the cases of *S*_*n*_ = 0 and *S*_*p*_ = 0, the maximum value appears 26.1 nm and 38.19 mA; for *S*_*n*_ = 0 and *S*_*p*_ = 1 × 10^7^ cm/s, the maximum is 28.7 nm and 38.15 mA; while for *S*_*n*_ = 1 × 10^2^ cm/s and *S*_*p*_ = 1 × 10^7^ cm/s, the maximum appears 7.25 nm and 32.46 mA. In fact, the bandgap of MoS_2_ deceases with increasing thickness, reducing the threshold of generating electronic-hole pairs^6^. In addition, MoS_2_ possesses excellent light absorption, thus the optical absorption increases with thickness and almost reach unit. Noticeably, Wong *et al*.^55^ reported that the ultrathin (<15 nm) vdW heterostructure can achieve the experimental absorbance more than 90%. Figure 4b plots the open-circuit voltage as a function of thickness. Clearly, the open-circuit voltage shows a slightly decrease with increasing thickness. Furthermore, the effect of surface recombination on the open-circuit voltage is obvious, while the back surface recombination has little effect.

In Fig. 4c, we can see that the PCE exhibits the similar tendency with short current density as the thickness increases. In detail, for the case of *S*_*n*_ = 0 and *S*_*p*_ = 0 the maximum PCE appears 15.05 nm and can be up to 24.76%; while for *S*_*n*_ = 1 × 10^2^ cm/s and *S*_*p*_ = 1 × 10^7^ cm/s, the maximum PCE is 6.6 nm and reaches 19.88%. Actually, this tendency is the joint effect of short-circuit current and open-circuit voltage. The carrier generation and collection enhance with increasing thickness due to decreasing bandgap and increasing optical absorption, thus the PCE increases rapidly in a few nanometers. Furthermore, with further increasing of thickness, the bandgap of MoS_2_ approaches to the bulk rapidly, and the diffusion length possesses a slightly decrease, leading to the lower PCE. The related experimental measurements of PCEs in MoS_2_/Si solar cells are about 5.23%^11^, 4.4%^12^, and 1.3%^13^, respectively. Moreover, the simulation on MoS_2_/Si possesses higher PCE of 12.44%^15^. In fact, device engineering such as surface contact, doping level and impurity density will depress the carrier collection and open-circuit voltage.

Remarkably, several experiments have proved that inserting suitable insulator at the interface is an effective way to improve the photoelectric conversion^18,19,56^. The intercalated insulator can suppress the static charge transfer, reduce leakage current and tune the Fermi level of MoS_2_, which suppresses interlayer recombination greatly and improves the performance of solar cells. For instance, the insert of SiO_2_ in bulk-like MoS_2_/Si heterostructure solar cell can effectively enhance the built-in field and promote the carriers separation, and achieve a high PCE of 4.5%^18^. Lin *et al*.^56^ found that the insert of h-BN into MoS_2_/GaAs can suppress the interlayer recombination, and the PCE increases from 4.82% to 5.42%. However, the insert layer will block carrier separation and collection when the thickness is greater than critical thickness^57^. Thus, a suitable buffer and optimal thickness of the insulator would be important to obtain high-performance solar cells.

Moreover, layered 2D material can form mixed-dimensional vdW heterostructure due to the weak interlayer interaction and elimination of dangling bonds^10,58^. Heterostructures consist of 0D n-MoS_2_ quantum dots and p-Si exhibits excellent light absorbing property, rectification behavior, and high photo responsivity and detectivity^59^. Furthermore, a hybrid vertical heterostructure by integrated 2D colloidal n-MoS_2_ nanocrystals on p-Si materials displayed high rectification ratio and high photo-to-dark current ratio^60^. Also, photodetectors of few-layer MoS_2_ integrated into amorphous Si possesses long-term stability^61^. Shin *et al*.^62^ optimized the photo response of MoS_2_/Si photodiode device by varying the MoS_2_ thickness, and found the excellent performance with a responsivity and detectivity of 76.1 A/W and 1012 Jones, respectively. Strikingly, mixed-dimensional vdW heterostructure suggests a considerable candidate in realistic fabrication and practice applications.

In summary, we explore the photoelectric properties of MoS_2_/Si in terms of bond relaxation method and DBP principle. It is found that the MoS_2_/Si exhibits type II band alignment with electrons at MoS_2_ layer while holes at Si layer, which is beneficial to improve the collection efficiency and photoelectric conversion. Our results show that the PCE of MoS_2_/Si improves as the thickness of MoS_2_ increases, and exhibits an obviously drops down with continuous increase due to infinite collection length. The excellent characteristics of MoS_2_/Si heterostructure demonstrate the great potential in 2D material-based solar cells.

## Method

### Atomic-bond-relaxation mechanism

Due to the absence of CN and the abrupt termination of bonding network at surface and edges, atoms at the surface and boundary will spontaneously shrink to the lowest energy state. In addition, at the interface formed by different materials, intrinsic strain will be generated at the interface due to mismatched lattice constants and coupling interaction at the interface. Considering the surface effect caused by under-coordinated and boundary atoms, the interface effect caused by lattice mismatch and interface coupling, as well as the strain caused by external stress or interface rotation, component doping and other factors, we develop the ABR method: the surface dangling bonds, interface mismatch, and the perturbation of external environment can be summed up in system thorough self-equilibrium strain. The lattice periodicity and Hamiltonian will change, leading to a series of physical quantities such as charge density and band gap are different from bulk. In general, the self-equilibrium strain of the system can be given according to $\partial U/V\partial {\varepsilon }_{ij}{|}_{{\varepsilon }_{ij}={\hat{\varepsilon }}_{ij}}$, where *U*, *V*, *ε*_*ij*_ (*i*, *j* = 1, 2, 3), respectively, represent total energy, volume and lattice strain.

### General approach on the photoelectric properties of 2D heterostructure

Here we assume that the photons with energy greater than bandgap generate one electron-hole pair, while the photons of lower energy produce no effect. Thus, the current density can express as:

2.1 $$G(x,\nu )=A(x,\nu )(1-R){f}_{w}{t}_{s}{Q}_{s}=\frac{2\pi (1-R){f}_{w}{t}_{s}}{{c}^{2}}{\int }_{{\nu }_{g}}^{\infty }A(v)\frac{{v}^{2}}{\exp (hv/{k}_{B}{T}_{s})-1}d\nu $$

where *t*_*s*_ is the probability that an incident photon produce a hole-electron pair, *f*_*w*_ denotes the geometrical factor^24^, *q* is the electronic charge and *T*_*s*_ is the temperature of sun.

Meanwhile, the carrier density satisfies the boundary conditions, by solving the differential equation under the boundary conditions, we have

22 $$\begin{array}{c}{I}_{n}=\frac{qG\alpha {L}_{n}}{{{\alpha }_{n}}^{2}{L}_{n}^{2}-1}\\ \,\,\,\,\times [\frac{{S}_{n}{L}_{n}/{D}_{n}+{\alpha }_{n}{L}_{n}-({S}_{n}{L}_{n}/{D}_{n}\,\cosh (d/{L}_{n})+\,\sinh (d/{L}_{n})){e}^{-{\alpha }_{n}d}}{{S}_{n}{L}_{n}/{D}_{n}\,\sinh (d/{L}_{n})+\,\cosh (d/{L}_{n})}-{\alpha }_{n}{L}_{n}{e}^{-{\alpha }_{n}d}]\end{array}$$

2.3 $${I}_{{scr}}=qG{e}^{-{\alpha }_{n}d}(1-{e}^{-{\alpha }_{n}{X}_{n}-{\alpha }_{p}{X}_{p}})$$

24 $$\begin{array}{c}{I}_{p}=\frac{qG{\alpha }_{p}{L}_{p}}{{\alpha }_{p}^{2}{L}_{p}^{2}-1}{e}^{-{\alpha }_{n}d-{\alpha }_{p}{X}_{p}}\\ \,\,\,\,\times [{\alpha }_{p}{L}_{p}-\frac{{S}_{p}{L}_{p}/{D}_{p}(\cosh (d/{L}_{p})-{e}^{-{\alpha }_{p}L})+\,\sinh (L/{L}_{p})+{\alpha }_{p}{L}_{p}{e}^{-{\alpha }_{p}L}}{{S}_{p}{L}_{p}/{D}_{P}\,\sinh (L/{L}_{P})+\,\cosh (L/{L}_{p})}]\end{array}$$

where *I*_*p*_, *I*_*scr*_ and *I*_*n*_ represent the current of quasi neutral *p* region, space charge region and quasi neutral *n* region, respectively. *d* = *D* − *X*_*n*_ represents the thickness of quasi neutral *n* region, and *L* = *D*_*Si*_ − *X*_*p*_ is the thickness of quasi neutral *p* region.

The equilibrium concentrations of electrons in MoS_2_ and holes in Si will have a change related to the difference in conduction band energy between MoS_2_ and Si. For the normalized radiative recombination current, the exponential dependence of the dissociation velocity on the band offset implies that^63^

2.5 $${J}_{0}={J}_{0}(SQ)\exp (-\Delta {E}_{{c}}/{k}_{B}T)$$

Moreover, for atomically thin and multilayer TMD heterostructures, the interlayer recombination dominates the carrier recombination process due to ultrafast separate of free carries at interface. The recombination can be obtained by a combination of Shockley-read-hall and Langevin recombination. Consider the discrepancy of different regions, the dark current density induced by recombination is

2.6 $${I}_{{\rm{0}}n}=\frac{q{D}_{n}{n}_{p0}}{{L}_{n}}\times [\frac{{S}_{n}{L}_{n}/{D}_{n}\,\cosh (d/{L}_{n})+\,\sinh (d/{L}_{n})}{{S}_{n}{L}_{n}/{D}_{n}\,\sinh ({L}_{n})+\,\cosh (d/{L}_{n})}]$$

2.7 $${I}_{0{scr}}=q(\frac{{n}_{p}{p}_{n}}{\tau ({n}_{p}+{p}_{n})}+B{n}_{p}{p}_{n}^{s})$$

2.8 $${I}_{0p}=\frac{q{D}_{p}{p}_{n0}}{{L}_{p}}\times [\frac{{S}_{p}{L}_{p}/{D}_{p}\,\cosh (L/{L}_{p})+\,\sinh (L/{L}_{p})}{{S}_{p}{L}_{p}/{D}_{p}\,\sinh (L/{L}_{p})+\,\cosh (L/{L}_{p})}]$$

Therefore, the current-voltage relationship can be modified:

2.9 $$I={I}_{sc}+{I}_{0}(1-\exp (\frac{qV}{{k}_{B}{T}_{c}}))$$

where ${I}_{sc}={I}_{p}+{I}_{{scr}}+{I}_{n}$ is the short current in the heterostructure, and ${I}_{0}={I}_{0p}+{I}_{0{scr}}+{I}_{0n}$ is the reverse saturation current. The open-circuit voltage (*V*_*oc*_) is obtained by solving Eq. (2.9) by setting *I* = 0, this leads to

2.10 $${V}_{oc}=\frac{{k}_{B}{T}_{c}}{q}\,\mathrm{ln}(\frac{{I}_{sc}}{{I}_{0}}+1)$$

Consequently, the limiting PCE is given by

2.11 $$\eta =\frac{{V}_{oc}{I}_{sc}FF}{{P}_{in}}$$

where $FF={z}_{m}^{2}/(1+{z}_{m}-{e}^{-{z}_{m}})({z}_{m}+\,\mathrm{ln}(1+{z}_{m}))$ is the fill factor, and the relationship between *V*_*oc*_ and *z*_*m*_ satisfies: ${V}_{oc}={k}_{B}{T}_{c}({z}_{m}+\,\mathrm{ln}(1+{z}_{m}))/q$, where *P*_*in*_ is the incident power.
